# Efficacy of Chinese Eye Exercises on Reducing Accommodative Lag in School-Aged Children: A Randomized Controlled Trial

**DOI:** 10.1371/journal.pone.0117552

**Published:** 2015-03-05

**Authors:** Shi-Ming Li, Meng-Tian Kang, Xiao-xia Peng, Si-Yuan Li, Yang Wang, Lei Li, Jing Yu, Li-Xin Qiu, Yun-Yun Sun, Luo-Ru Liu, He Li, Xin Sun, Michel Millodot, Ningli Wang

**Affiliations:** 1 Beijing Tongren Eye Center, Beijing Tongren Hospital, Capital Medical University, Beijing, China; 2 School of public health, Capital Medical University, Beijing, China; 3 Anyang Eye Hospital, Anyang, Henan Province, China; 4 Chinese Evidence-based Medicine Center, West China Hospital, Sichuan University, Chengdou, China; 5 Clinical Research and Evaluation Unit, West China Hospital, Sichuan University, Chengdou, China; 6 Department of Clinical Epidemiology and Biostatistics, McMaster University, Hamilton, Canada; 7 School of Optometry and Vision Sciences, Cardiff University, Cardiff, United Kingdom; Medical College of Soochow University, CHINA

## Abstract

**Purpose:**

To evaluate the efficacy of Chinese eye exercises on reducing accommodative lag in children by a randomized, double-blinded controlled trial.

**Methods:**

A total of 190 children aged 10 to 14 years with emmetropia to moderate myopia were included. They were randomly allocated to three groups: standard Chinese eye exercises group (trained for eye exercises by doctors of traditional Chinese medicine); sham point eye exercises group (instructed to massage on non-acupoints); and eyes closed group (asked to close their eyes without massage). Primary outcome was change in accommodative lag immediately after intervention. Secondary outcomes included changes in corrected near and distant visual acuity, and visual discomfort score.

**Results:**

Children in the standard Chinese eye exercises group had significantly greater alleviation of accommodative lag (-0.10D) than those in sham point eye exercises group (-0.03D) and eyes closed group (0.07D) (P = 0.04). The proportion of children with alleviation of accommodative lag was significantly higher in the standard Chinese eye exercises group (54.0%) than in the sham point eye exercises group (32.8%) and the eyes closed group (34.9%) (P = 0.03). No significant differences were found in secondary outcomes.

**Conclusion:**

Chinese eye exercises as performed daily in primary and middle schools in China have statistically but probably clinically insignificant effect in reducing accommodative lag of school-aged children in the short-term. Considering the higher amounts of near work load of Chinese children, the efficacy of eye exercises may be insufficient in preventing myopia progression in the long-term.

**Trial Registration:**

ClinicalTrials.gov NCT01756287

## Introduction

Myopia is a public health problem worldwide, especially in Asian countries such as Singapore, Japan and China[1]. Myopic retinopathy occurs in about 40% of highly myopic eyes[2] and has become the second leading cause of blindness and low vision in Chinese, ranging from 7.7%–32.7%[3,4]. Myopic retinopathy is also one of the most common causes (6%) of visual impairment and blindness in European-derived populations[5]. Therefore, any kind of method designed for preventing or slowing myopia progression deserve scientific evaluation on its efficacy and safety for controlling myopia.

Chinese eye exercises, a kind of massage around periocular acupoints, were originated and imbued with theories of traditional Chinese medicine[6]. Since 1963, the Chinese government has endorsed these exercises in the belief that they protect vision and may prevent myopia in children. They have become a community ritual and a living habit of students in primary and middle school in China for half a century. Meanwhile, myopia prevalence in Chinese children has increased remarkably and reached epidemic levels (30.1%~78.4%) in recent decades[7,8]. Therefore, it would not appear that these eye exercised play a critical role in preventing myopia[9] or alleviate eyestrain. Although it was reported that doing Chinese eye exercises might not related to the prevalence of myopia in children,[10] some argue that the prevalence might have been much greater had children not performed the exercises. Some studies showed that about 90% of Chinese children didn’t perform these exercises correctly although they did them everyday [11,12]. Most Chinese children couldn’t find the exact periocular acupoints and didn’t have accurate pressure and manipulation skills for the exercises[11,12].

Whether poor manipulation quality of Chinese eye exercises or the exercises itself influence its effect on preventing myopia progression remains to be proven. To date, there has not been a randomized controlled trial (RCT) which has investigated whether Chinese eye exercises are effective in preventing myopia, or at least alleviating eyestrain in children if the exercises are performed or manipulated correctly. After communication with education officers, school teachers and parents, it was made clear that it would be impossible to perform a RCT with long-term outcome such as myopia progression by allocating some children to not performing Chinese eye exercises for years.

A recent study showed that Chinese eye exercises had a modest effect on relieving near vision symptoms among Chinese children.[13] Shih et al. found that Qi-Qong ocular exercises could improve the accommodative amplitude and accelerate the accommodative response slightly,[14] which might be the underlying mechanism for relieving near vision symptoms. However, it is still unclear how these exercises could physiological affect myopia development. There is a paucity of convincing evidence: In one study it was found that stimulation of auricular acupoints enhanced the effect of atropine in controlling myopia. One possible mechanism which could account for a reduction in myopia development or progression is accommodative lag. It is known that there is an increased lag of accommodation in myopic subjects[15–18], although some authors did not find this association[19,20]. However, the crucial question remains whether accommodative lag increases before or after the onset of myopia. There seems to be more evidence supporting the hypothesis that it occurred before the onset of myopia[21–23] than the reverse[24]. Thus, short-term outcomes associated with myopia or eyestrain, such as accommodative lag, could be considered to evaluate the Chinese eye exercises.

The aim of this double-blind, randomized controlled trial was to investigate whether Chinese eye exercises reduce accommodative lag of school-aged children in the short-term. Corrected visual acuity at distant and near and visual discomfort score were also evaluated to see whether Chinese eye exercises simultaneously affect subjective visual function. In the affirmative, the exercises might be worth popularizing with strict training, rather than the current condition with poor manipulation quality in most children, to prevent myopia progression or at least alleviate eyestrain in children.

## Methods

The protocol for this trial and supporting CONSORT checklist are available as supporting information; see S1 CONSORT Checklist and S1 Protocol.

This trial was conducted at a middle school in Anyang city, Henan province, central China, where we have established two cohorts of schoolchildren to investigate risk factors and possible interventions for myopia[25,26]. Inclusion criterias were visual acuity of 20/20 or better in each eye, spherical equivalent ranging from +0.50D to-6.00 D, astigmatism less than-1.50D in each eye, anisometropia less than 1.0 D, and no history of any ocular or systemic diseases. Exclusion criteria were currently using any other interventions to control myopia progression, or unable to cooperate with exercise training and ocular examinations. Oral consent was obtained from all participants and written informed consent was obtained from their parents before enrollment. The trial has been approved by the ethics committee of Beijing Tongren Hospital and registered in ClinicalTrials.gov (NCT01756287).

Based on data collected in a preliminary set of measurements, we anticipated a mean difference of 0.18±0.16 D in accommodative lag among the three groups, and determined to have a power of 90% in statistical test and two-sided α = 0.05 to detect the difference in treatment effect[27]. Assuming an estimated loss to follow-up of 20%, we calculated that we should enroll at least 180 children. Eligible children were randomly allocated in a ratio of 1:1:1 to three groups: standard Chinese eye exercises group, sham point eye exercises group and eye closed group. The participant recruitment was conducted from January 2013 to November 2013 and follow-up was finished one month later.

**Fig 1:**
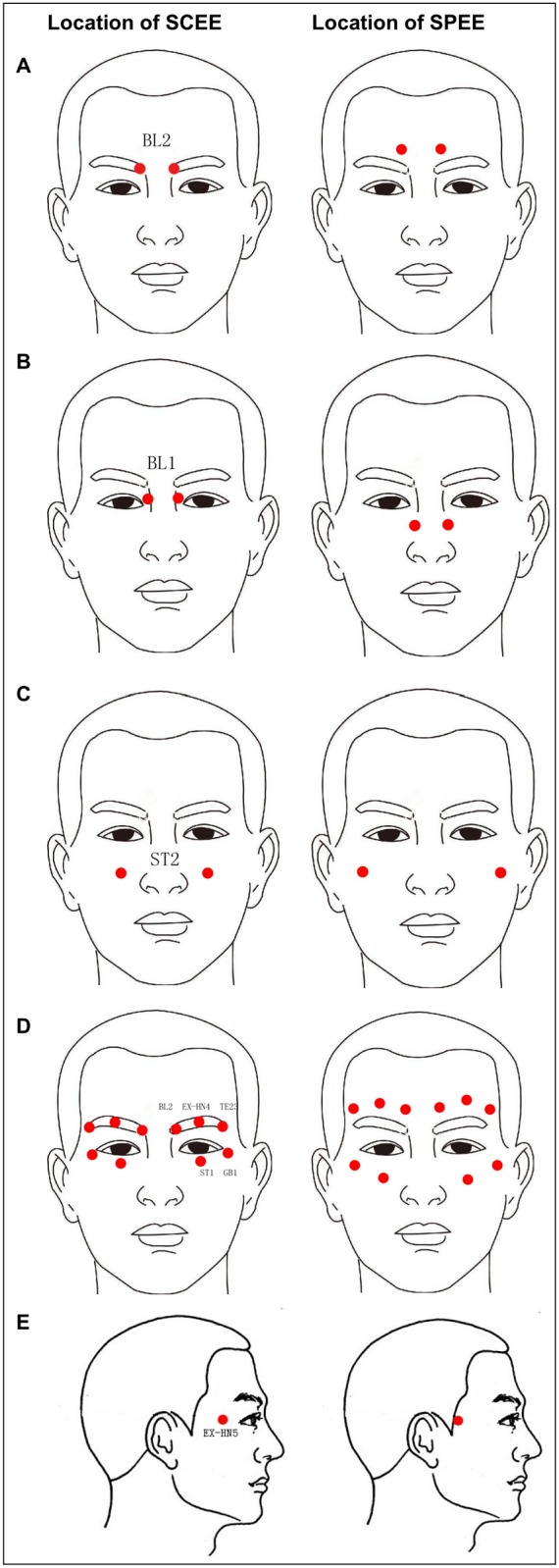
Locations of acupoints of standard Chinese eye exercises and sham point eye exercises.

An expert committee composed of Ophthalmologists and doctors of traditional Chinese medicine was responsible for displaying accurate acupuncture points of each intervention and assessing performance quality of exercises for each participant. In the first group sixteen acupuncture points (bilateral BL2, BL1, ST2, EX-HN4, EX-HN5, TE-23, ST1 and GB1) of four sections of exercise were chosen for the standard Chinese eye exercises group according to the WHO Standard Acupuncture Point Locations in the Western Pacific Region[28]. In the second group sham point eye exercises group, acupuncture points were designed to be located at places without acupoints and 2 cm away from those of standard Chinese eye exercises (Fig. 1, Table 1). In the third group with only closed eyes, no acupuncture points were chosen.

**Table 1:** Locations of Acupoints and Manipulation Methods Used in the Standard Chinese Eye Exercises and Sham Point Eye Exercises Group.

Acupoint Code (Name in Chinese)	Manipulation	Location of standard Chinese eye exercises	Location of sham point eye exercises
1-BL2 (cuanzhu)	Press in circle for 1min	In the supraorbital notch and at the median end of the eyebrow	2cm above BL2
2-BL1 (jingming)	Press in circle for 1min	In the depression slightly above the inner canthus	2cm below the BL1
3-ST2 (sibai)	Press in circle for 1min	Directly below the pupil, in the depression of the infraorbital foramen	2cm lateral aside the ST2
4-EX-HN5 (taiyang)	Press in circle for 30s	Flat part at each side of the forehead	2cm lateral aside the EX-HN5
TE-23 (sizhukong), EX-HN4 (yuyao), BL2 (cuanzhu),GB1 (tongziliao), ST1 (chengqi)	Scrape the eyebrows for 30s	On the orbit	2cm lateral aside the orbit

One investigator first demonstrated the procedures of each eye exercises and explained the details. Participants were then asked to practice respective exercises under the instruction for at least three times. During this period, instructors repeatedly corrected inappropriate performance until the participants performed the exercises accurately. Finally, two independent assessors assessed and scored all participants’ performance quality of exercises. The assessment consisted of 40 items including location, force, scope, and frequency of massage with 1 score of each item. On the basis of total score of 40, scores greater than 30/40 and differences between two judges less than 5 points were defined as qualified. On the following day, interventions were administered at 7:00 a.m. before morning reading class. Then every 6 participants were asked to perform the exercises once for 5 minutes at the same time.

Random sequence was generated by one independent statistician. Stratified random sampling with cycloplegic autorefraction (spherical equivalent) was used to three layers; emmetropia (+0.50D~ -0.50D), low myopia (-0.50D~ -3.00D) and moderate myopia (-3.00D~ -6.00D). For each stratum, we applied a block of 6 for randomization to ensure equal allocation of participants in treatment groups. We used opaque assignment envelopes with consecutive numbers to conceal allocation.

Participants, outcome assessors and statistician were all masked to the interventions. One day prior to the interventions, all participants were first taught with a didactic lesson on how to protect their eyes. Then they were separated to learn about standard Chinese eye exercises, sham point eye exercises or eye closed by one investigator. All participants were told to learn a type of new eye exercise. Communication with each other or interaction with the examiners was strictly limited to reduce non-specific effects[29]. Examiners were asked to be absent when children practiced and performed respective eye exercises back to back. All data of outcome measures were analyzed by one independent statistician who was masked to the allocation.

Primary outcome was mean difference in accommodative lag before and immediately after the intervention. A masked assessor measured accommodative response with an open-field autorefractor (Grand Seiko, WAM-5500, Japan) during binocular viewing. Participants were instructed to focus on a distant letter chart at 4m for 5 seconds and a near letter chart at 33 cm for another 5 seconds, and were asked to fixate on 20/100 letters. Accommodative lag was equal to the difference between effective accommodative demand and accommodative response according to equations published previously[30].

Secondary outcomes included corrected near and distant visual acuity measured with logarithmic visual acuity chart (Precision Vision, USA) at 40 cm and 4 meter, respectively. Visual discomfort score was assessed by a self-report survey developed by Conlon[31] including accommodative function, sensitivity to light, eye strain and common symptoms. Scores range from 0 to 100 and smaller scores indicating less visual discomfort. An interviewer-administered questionnaire was completed by the children to collect the time spent on near work[32].

Mean differences before and immediately after intervention was calculated. Only the results of the right eye were used for analysis. If the values were normally distributed, analysis of Covariance (ANCOVA) and post-hoc tests (S-N-K method) were used. Otherwise, nonparametric test (rank sum test) was used. Chi-square test was used to compare categorical outcomes. All P-values were 2-sided and considered statistically significant when less than 0.05 except for 0.017 corrected for post-hoc tests (there were 3 combinations of paired-comparisons from three groups).

## Results

**Fig 2:**
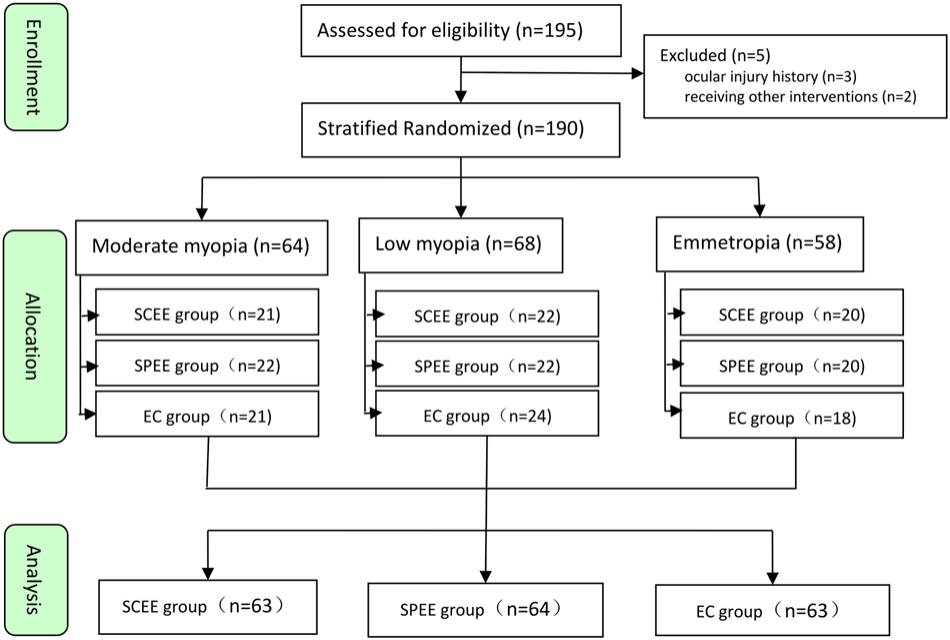
Flow of participant through trial.

We recruited 195 grade 7 students and excluded 5 (3 for a history of ocular injury and 2 for receiving other interventions). Of 190 eligible participants, 63 were randomly assigned to the standard Chinese eye exercises group, 64 to the sham point eye exercises group, and 63 to the closed eye group (Fig. 2). No participant was lost in each group. Mean age was 12.62 ± 0.56 years, and 50% were male. Baseline characteristics were balanced among the three groups (Table 2).

**Table 2:** Baseline Characteristics of Study Participants in Three Groups.*

Characteristic	Standard Chinese eye exercises group（n = 63）	Sham point eye exercises group (n = 64)	Eye closed group (N = 63)	F or χ^2Ψ^	P
Gender n (male: female)	27:36	32:32	36:27	1.406^**Ψ**^	0.276
Mean age (± SD)	12.60±0.49	12.70±0.64	12.57±0.59	0.832	0.437
Spherical equivalent (diopter)	-1.98±1.80	-2.17±1.91	-2.11±1.84	0.140	0.869
Astigmatism (diopter)	-0.40±0.39	-0.39±0.40	-0.46±0.74	0.269	0.764
Corrected distant visual acuity	-0.01±0.11	-0.02±0.10	-0.01±0.10	0.624	0.537
Corrected near visual acuity	-0.06±0.09	-0.05±0.08	-0.03±0.09	2.194	0.114
Accommodative response (diopter)	1.95±0.50	1.96±0.44	1.81±0.44	2.224	0.111
Visual discomfort score	6.1±7.0	5.6±6.4	7.0±6.8	0.690	0.503
Near work (hours/week)	32.92±16.11	32.36±20.31	36.03±17.01	0.750	0.475

**Fig 3:**
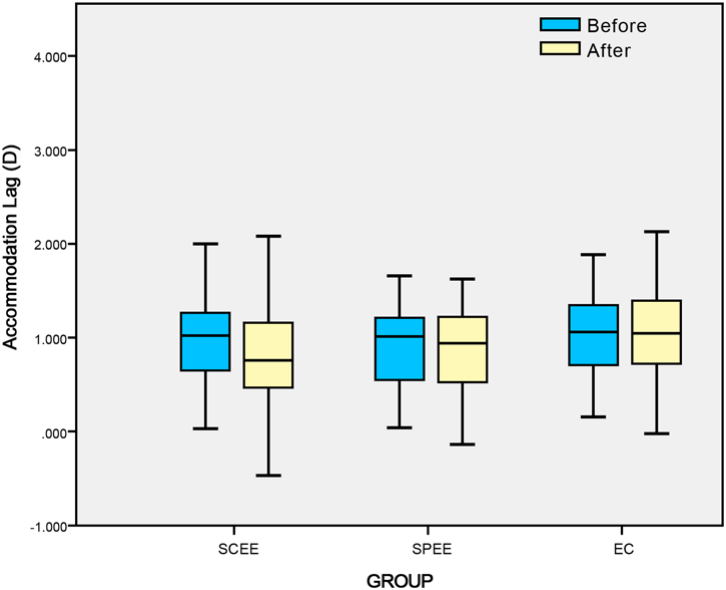
Distribution and mean of accommodative lag before and after intervention.

* Plus—minus values are means ±SD unless otherwise noted;

Fig. 3 shows the scatter plot and mean of accommodative lag before and immediately after the interventions. Table 3 shows that participants in the standard Chinese eye exercises group had a significant improvement in accommodative lag (-0.10 D) than those in the eye closed group (0.07 D) and the sham point eye exercises group (-0.03 D) (P = 0.04). Post-hoc tests showed that standard Chinese eye exercises group was significantly different from sham point eye exercises group (P = 0.014) and eye closing group (P = 0.007). There was no significant difference between sham point eye exercises group and eye closing group (P = 0.091). The alleviation in accommodative lag occurred in 54.0% of participants in the standard Chinese eye exercises group, which was significantly higher than that in the sham point eye exercises (32.8%) and the eye closed group (34.9%) (P = 0.03).

**Table 3:** Changes in accommodative lag and other secondary outcomes before and immediately after different eye exercises.

Outcomes	Group	Median (lower ~upper quartile)	F	P	Improved (%)	χ^2^	P
Accommodative lag	SCEE	-0.10 (-0.37~+0.18)	6.245	0.044	34(54.0%)	7.12	0.028*
	SPEE	-0.03 (-0.26~+0.13)			21(32.8%)		
	EC	0.07 (-0.20~+0.24)			22(34.9%)		
Corrected distant VA	SCEE	-0.04 (-0.08~0.00)	3.284	0.194	41(65.1%)	2.50	0.287
	SPEE	-0.02 (-0.06~0.00)			33(51.6%)		
	EC	-0.02 (-0.04~0.00)			35 (55.6%)		
Correct near VA	SCEE	-0.02 (-0.08~0.00)	1.989	0.370	40(63.5%)	3.922	0.141
	SPEE	-0.02 (-0.06~+0.02)			36(56.3%)		
	EC	-0.02 (-0.06~0.00)			32(50.8%)		
Visual discomfort score	SCEE	-1.00 (-3.00~+1.00)	2.382	0.304	43(68.3%)	1.73	0.132
	SPEE	-1.00 (-4.00~0.00)			49(76.6%)		
	EC	0.00 (-2.00~1.00)			42(66.7%)		

SCEE: standard Chinese eye exercises; SPEE: sham point eye exercises; EC: eyes closed;

*Post-hoc tests: SCEE group vs. SPEE group, χ^2^ = 12.430, P = 0.014; SCEE group vs. EC group, χ^2^ = 13.995, P = 0.007; SPEE vs. EC group, χ^2^ = 0.015, P = 0.901

There were no significant differences between before and after intervention in the findings of near and distant visual acuity, visual discomfort score and the proportions of improvement for these outcomes (Table 3).

## Discussion

Our study showed that Chinese eye exercises significantly alleviate accommodative lag of children in the short-term compared to sham point eye exercises and closed eyes, which supported the findings of a previous study conducted in Taiwan that Qi-Qong ocular exercise could improve the accommodative amplitude and accelerate the accommodative response slightly. Changes in corrected near and distant visual acuity and visual discomfort score, however, were insignificant in our study. The alleviation in accommodative lag found in this study, although it would not appear to be clinically meaningful, might implicate a potential to delay myopia progression in children in the long run through brief periods of increasing myopic defocus when doing near activities.

Animal experiments have shown that brief periods of myopic defocus could prevent myopia caused by daylong period of hyperopic defocus[33,34], and eyes can rapidly recognize the sign of defocus[35], especially myopic defocus[36]. Applying these findings to children, since Chinese eye exercises alleviate accommodative lag, eyes would accommodate more and suffer from more myopic defocus when doing near activities. Thus, Chinese eye exercises may slow myopia progression in children by frequent interruption of constant hyperopic defocus with brief periods[36].

Some animal experiments showed that accommodation might not be important in the development of myopia[37]. Mutti et al. found no significant association between accommodative lag and myopia progression in children[24,38,39]. However, other studies showed that accommodative lag was associated with myopia progression in children[22,23]. and accommodative lag was also independent predictors of myopia progression in young adults.[17] In addition, multifocal lenses, designed to reduce accommodative demand and then eliminate accommodative lag at near, could reduce myopia by a small but statistically significant amount, especially in Asian children[40]. To the best of our knowledge there does not seem to be an investigation evaluating how much associated lag is essential to have an impact in reducing myopia progression.

In the present study, the magnitude of alleviation of accommodative lag was about 0.10D, slightly greater than the reported intertest repeatability (0.07D) of noncycloplegic measurements with WAM-5500[41]. Thus, changes in accommodative lag in our study might be biased from the noise of the instrument itself when performing noncycloplegic measurements on two different occasions. Moreover, we evaluated the effect of Chinese eye exercises for only 5 minutes which was a small dosage of stimulation. Chinese eye exercises are usually performed by Chinese children twice daily. This frequency might not be effective in interrupting hyperopic defocus caused by long-term near work, which requires further and long-term study.

As we expected, there were no significant changes in visual acuity and visual discomfort score among the three groups. With only one time of exercises, it would be unreasonable to find significant changes in visual function. However, it should be noted that standard Chinese eye exercises resulted in greater quantitative (-0.04) and qualitative (65.1%) improvement in corrected distant visual acuity compared to the sham point eye exercises (-0.02 and 51.6%) and closed eye groups (-0.02 and 55.6%); and greater qualitative (63.5%) improvement in corrected near visual acuity compared to the sham point eye exercises (56.3%) and closed eye groups (50.8%). The improvement in distant visual acuity might be a consequence of alleviation of accommodative lag since increased accommodation lag was reported to be associated with decreased visual acuity[20].

In a previous study, 5 adults exposed to taxing visual tasks were tested for accommodation (using laser optometer) and visual acuteness at high frequency (critical flicker fusion apparatus) before and after 4 weeks’ performance of Chinese eye acupressure practice[6]. They reported no significant changes in accommodation and visual acuteness and attributed the negative results to a small sample, no control, and the older age of participants (27~30 years old). In the present study, 46.0% of children in the standard Chinese eye exercises group showed no improvement in accommodative lag. This indicated that children had varied responses to Chinese eye exercises. Many factors such as genetic factors, psychological factors, sensitivity to acupoint and degree of myopia might explain these different responses[42]. Recently, a randomized controlled acupuncture trial showed that non-acupuncture factors such as participants’ expectations would confound the results of acupuncture therapy by activating multiple brain areas[42]. To minimize the influence of psychological expectations, we informed the participants in all groups that they were invited to learn different types of eye exercises.

The precise mechanism by which Chinese eye exercises influences accommodative lag in still unclear. Massage on BL2, GB14, TE 23, Ex1, ST1, GB20, LI4, LI11 and GV23 may increase chorioretinal[43] and regional cerebral blood flow[44]. In addition, acupuncture may activate visual cortex[45] and cause metabolic changes in the central nervous system[46]. Acupuncture trial showed that massage on BL2 and ST1 may promote tear stability and secretion, increase tear lactoferrin level, and affect retrobulbar circulation and intraocular pressure (IOP)[47]. EX-HN5 may have a therapeutic effect on dizziness and headaches[48]. It is hypothesized that Chinese eye exercises may increase blood flow to the eyeball, enhance parasympathetic-driven responses in the ciliary muscle by stimulation on ocular region or visual cortex, and thereby affect the accommodation of the eyes.

Our results might explain the findings of previous trial that stimulation of auricular acupoints enhanced the effect of 0.25% atropine in controlling myopia in school-aged children[49].[42] Auricular acupoints used in that trial are related to the eye from the viewpoint of the meridians of traditional Chinese medicine, and are functionally similar to periocular acupuncture points used in the present trial. If the short-term improvement of accommodative lag by massage on eye-related acupuncture points could be cumulative and lasting, becoming clinically significant, myopia might be controlled in the long-term. Whether the current dose of eye exercises can lead to a cumulative effect of alleviating accommodative lag remains unproven and warrants further research, especially as they are administered twice daily for 5 minutes each, in most schools in China, and about 90% of Chinese children do not perform them correctly [11,12].

Although the results obtained in this trial are promising, some interesting questions remain unanswered. First, Chinese eye exercises are not a strong stimulation compared to acupuncture. How long could the improvement in accommodative lag obtained in this trial last remains to be determined. Second, its long-term effect on preventing myopia development or slowing myopia progression remains to be investigated. However, further research needs approval from the government since the parents and teachers do not allow a long-term cessation of Chinese eye exercises in children. If the findings of this trial are available for the public, it may promote future trials. Third, we did not measure tear function, blood circulation and subjective accommodation due to limited time after the interventions. We chose right eyes for measuring outcomes across all participants for operational reasons. Although there is a risk of selective outcome measurement, we anticipate that its impact on the effect estimates is trivial. Forth, only 54.0% of children had alleviation of accommodative lag. This might be due to the difficulty of performing the exercises correctly although we had trained the children carefully.

Other evidence of Chinese eye exercises on myopia is still worthwhile. First, in other areas with high prevalence of myopia such as Singapore, Hong Kong, Taiwan, Japan and Korea, acupuncture point based eye exercises were not as popular as in China. The correlation between the use of eye exercises and myopia prevalence could be tested by comparison among these areas. Secondly, although a long-term randomized controlled trial is difficult to be performed now, it would be meaningful to evaluate the differences in progression and incidence of myopia among children with varied skill and quality of performing Chinese eye exercises, which need a longitudinal study of myopia status in children such as the cohort from the Anyang Childhood Eye Study.[32] Both of these two approaches will provide further evidence on this issue and many confounding factors need to be considered during the evaluation.

## Conclusions

In summary, our preliminary findings indicate that Chinese eye exercises transiently alleviate accommodative lag in children at a statistically but probably clinically insignificant level. The efficacy of Chinese eye exercises may be insufficient in preventing myopia progression in the long-term considering the higher near work load of Chinese children. Time spent in near work of children in this trial were 32.36~36.03 hours/week which was much higher than that of Australian children of the same age (27.4 hours/week)[50]. Further long-term follow-up and other evidence are warranted to evaluate the necessity of continuously generalizing these exercises in China.

## Supporting Information

S1 CONSORT Checklist (DOC)

S1 Protocol Trial Protocol in Chinese. (DOCX)

S2 Protocol English translation of the original protocol. (DOCX)
