# Supplementary material for: Efficacy of Chinese Eye Exercises on Reducing Accommodative Lag in School-Aged Children: A Randomized Controlled Trial
Source: PLoS One. 2015 Mar 5;10(3):e0117552. doi: 10.1371/journal.pone.0117552 (PMC4350838; doi:10.1371/journal.pone.0117552)
Supplement: S1 Protocol — (DOCX) [file pone.0117552.s002.docx]

**眼保健操对青少年眼调节迟滞的影响：**

**随机对照试验**

**操作手册**

**（第三版）**

北京同仁医院

安阳眼科医院

“安阳儿童眼病研究”课题组 编制

2012年9月

**第一章 总则**

1. **研究背景**

我国是世界上的近视眼大国。近年来，我国青少年近视患病率持续上升，近视的程度越来越高。如何防治或延缓青少年近视的进展已经成为了社会各界急盼解决的问题。

眼保健操是由北京医学院体育教研组刘世铭主任于1963年创立的，是根据中医推拿、经络理论，结合体育医疗综合而成的按摩法，它通过对眼部周围穴位的按摩，使眼内气血通畅，改善神经营养，以达到消除睫状肌紧张或痉挛的目的。当今，眼保健操已经成为我国学生在校学习期间每天实施的作为缓解眼疲劳、保护学生视力的重要措施。然而近年来，我国青少年近视患病率仍然逐年上升，眼保健操遭到来自学生、家长、老师多方质疑，社会上“眼保健操无用，应该取消”的呼声不断。眼保健操在防治青少年近视发生和发展中或者在缓解青少年视疲劳中究竟是否有效？目前国内外仍缺少能提供有力科学证据的试验研究。

国内已有的评估眼保健操的研究以观察性研究为主^[^[^1-6^](#_ENREF_1)^]^，设有对照的研究较少^[^[^7-10^](#_ENREF_7)^]^，极少的标记为为随机对照试验的研究^[^[^11-13^](#_ENREF_11)^]^也未按照随机对照试验的国际报告规范CONSORT ^[^[^14^](#_ENREF_14)^,^ [^15^](#_ENREF_15)^]^来报告研究结果，因此其所提供证据的可信度不高。此外，目前关于眼保健操效果的研究其评估指标多为视力^[^[^1^](#_ENREF_1)^,^ [^6^](#_ENREF_6)^,^ [^9^](#_ENREF_9)^,^ [^10^](#_ENREF_10)^]^，较少有采用屈光度^[^[^16^](#_ENREF_16)^]^和眼轴长度等客观指标。而对于眼科专业来讲，纵观国际上经典的近视干预措施研究，屈光度和眼轴长度是衡量近视是否稳定或进展的金指标^[^[^17^](#_ENREF_17)^,^ [^18^](#_ENREF_18)^]^。而且，由于青少年的调节力较强，大量的视近学习工作使其睫状肌长期处于紧张痉挛状态，小瞳验光所得到的屈光度误差较大^[^[^19^](#_ENREF_19)^]^，屈光度应当以散瞳验光的结果为准。

眼保健操的评估也可以考虑短期评估指标，但应当以稳定性、可重复性较好，并且能通过客观仪器进行测量的指标为佳。国内外的大量研究发现调节与近视的发生发展密切相关^[^[^20^](#_ENREF_20)^,^ [^21^](#_ENREF_21)^]^，调节迟滞、调节反应和调节灵活度等指标可通过客观方法测量，是评估近视干预措施效果的较为理想的短期指标。调节也是评估视疲劳的重要指标^[^[^22^](#_ENREF_22)^,^ [^23^](#_ENREF_23)^]^，眼保健操则被认为至少可缓解青少年视疲劳^[^[^24^](#_ENREF_24)^,^ [^25^](#_ENREF_25)^]^。因此，通过测量调节参数来评估眼保健操的短期效果也是较为可行的客观方法。近视力^[^[^26^](#_ENREF_26)^]^和视疲劳评分^[^[^27^](#_ENREF_27)^]^是与调节参数密切相关的主观指标，可以作为短期观察效应指标的有益补充。

调节是指人眼能把外界不同距离的物体反射的光线经由眼球屈光系统清晰聚焦于视网膜的能力。调节反应的测量方法通常采用动态检影法和双眼交叉柱镜DCC法，这两种方法需要被检查者的充分理解以及配合，尤其是后者主观性较强，因此在青少年中较难开展，其结果的可信度和重复性也不高。双眼开放视野自动验光仪为一种客观的调节检查方法，在国外被广泛应用于调节反应的研究中，能够较为客观灵敏地检测眼调节力的变化，同时还能够测量瞳孔的大小。瞳孔大小和调节密切相关，如果眼保健操能够放松调节，则理应也能够影响瞳孔大小。眼保健操能否在短期内影响青少年近视眼的调节，对长期预防近视发展是否有影响，眼保健操的有效性和与穴位按摩的准确度是否有关，这一系列的问题值得我们深入研究。我国目前尚缺乏针对此问题的随机对照试验，

因此，本研究拟通过随机对照双盲试验评估眼保健操前后青少年的眼调节、近视力、视疲劳评分和瞳孔大小的变化，以探究眼保健操的有效性。

1. **研究目的**

分析眼保健操对青少年近视眼调节的影响。

1. **研究假说**

规范标准化的眼保健操能够有效改善青少年近视眼疲劳和放松调节。

1. **研究内容**
2. 分析青少年近视眼在眼保健操前后的调节参数变化；
3. 分析青少年近视眼在眼保健操前后的近视力变化；
4. 分析青少年近视眼在眼保健操前后的视疲劳评分变化；
5. **研究方案**
6. 试验设计：随机对照双盲试验，区组设计。

（试验组：对照组一：对照组二=60：60：60）

1. 试验日期：2012年9月。
2. 试验地点：河南省安阳市师院附属中学，检查室。
3. **研究对象**
4. 研究对象：初二年级学生
5. 样本量：180人
6. 纳入标准

-双眼外观正常，无眼外伤、斜视（隐斜视除外）、弱视或任何病理性改变；

-双眼中任何一眼的最佳矫正视力可达1.0；

-中度近视，即双眼中任何一眼的等效球镜为+0.50D至-6.0D；

-双眼任何一眼的散光小于-1.50D；

-双眼屈光参差小于1.0D；

-本人及家长同意参加本调查并签署知情同意书；

3. 排除标准：

-目前正在接受其他近视治疗（针灸，按摩，仪器，药物，耳针等）；

-检查当天身体不适者（感冒，头疼）；

4. 被调查者的退出或终止试验的标准

在下列情况下研究对象将被从研究中剔除或终止随访：

- 干预过程中出现严重不适的学生；

- 不依从者。如：学生或家长严重违反试验规则，不能按照分组执行干预；

- 由于各种原因，学生或家长不愿继续参加本研究；

1. **观察指标**

1、主要结局指标：调节迟滞。

2、次要结局指标：瞳孔直径、近视力、视疲劳评分。

1. **干预措施**

●试验组： 规范培训后的眼保健操；

试验前一日，**在对照组学生不在场的情况下，**两位试验组学生将由一位专业中医培训正确的眼保健操做法，直至考察操作合格。

**培训内容：**包括**示范**和**操作培训**两部分。培训时间不少于半小时。

**示范**：由中医示范与眼保健操教学图结合讲解标准眼保健操取穴位置、按摩手法、按摩力度、按摩范围；重点讲解取穴方法，如何确认取穴是否正确。

1. 揉天应穴：天应穴位于眉头正下方一点点，靠眼眶内侧的位置，在眉头下方的位置附近用指端试按，有一个小凹陷处，用一点力，明显感到发酸的位置就是穴位所在。按揉用拇指指端，揉法为划圈，一拍画一个圈，范围不要超过两个指尖；其他四指弯曲扶在前额上。按揉的过程中有酸胀感说明是正确的。
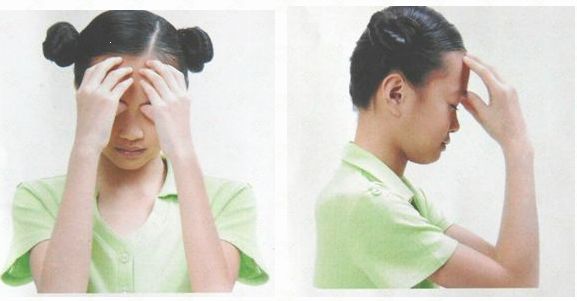

2. 挤按睛明穴：睛明穴位于鼻梁两侧眼角半分的地方。用两食指按在睛明穴上，先向下按后向上挤，一按一挤为一拍。范围不要超过两个指尖。挤按时是**用手指按压摩擦骨头**的感觉，不是捏皮肤。挤按的过程中有酸胀感说明是正确的。
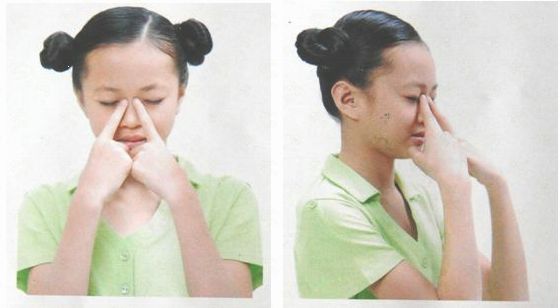

3. 揉四白穴：先以两手中指和食指并拢伸直，不要分开，然后中指指肚轻靠两侧鼻翼最宽处，食指尖所按之处即四白穴，按下时有酸胀感。这一节很多同学会大面积画圈，这样是不对的，按揉四白穴时面积不要超过两个指尖。按揉的过程中有酸胀感说明是正确的。
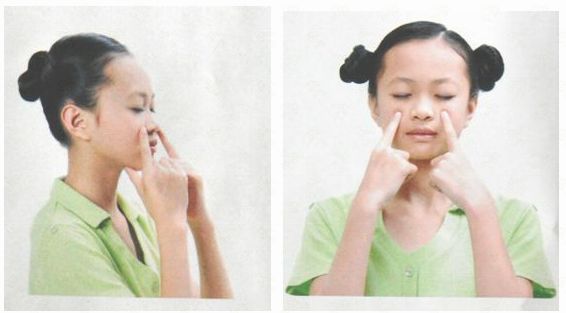

4. 按揉太阳穴轮刮眼眶：首先用大拇指找到太阳穴，在外眼角与眉梢之间向后大约一寸的地方，这里的凹陷即为太阳穴，轻揉太阳穴，力度有酸胀感即可，不能大力按揉。轮刮眼眶位置为上下眼眶骨性突出部分，轮刮长度覆盖整个眼眶。轮刮一圈是四拍，再用拇指肚按揉太阳穴四拍。按揉的过程中有酸胀感说明是正确的。
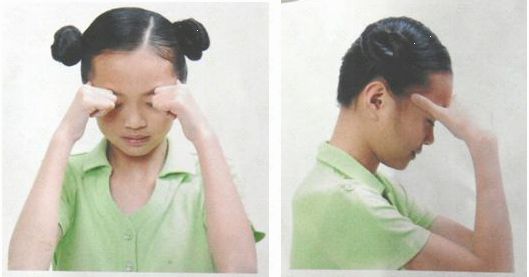


**操作培训：**

一、由学生分节做眼保健操，每节做八拍；

二、在每一节的操作过程中，如果学生出现错误，由培训人员及时纠正并按揉学生的正确穴位，讲解正确穴位的找法；

三、在每一节的操作结束后，由培训人员在学生的穴位上按揉找穴，按揉出酸胀感，加深学生印象，强调按揉过程中有酸胀感说明是正确的。

四、纠正一遍后，最后再由学生分解动作完成眼保健操，检查是否正确。重点记录学生做错的动作，培训后再次强化教学。

**合格标准：**详见评分标准，满分40分，由两位培训人员分别独立评估。要求每位学生都能够独立、正确完成两遍眼保健操。两位培训人员评估学生分数在30分以上，且两者评分差值不超过5分为合格。如果不合格，需再次培训，直到合格为止。

眼保健操的局部穴位
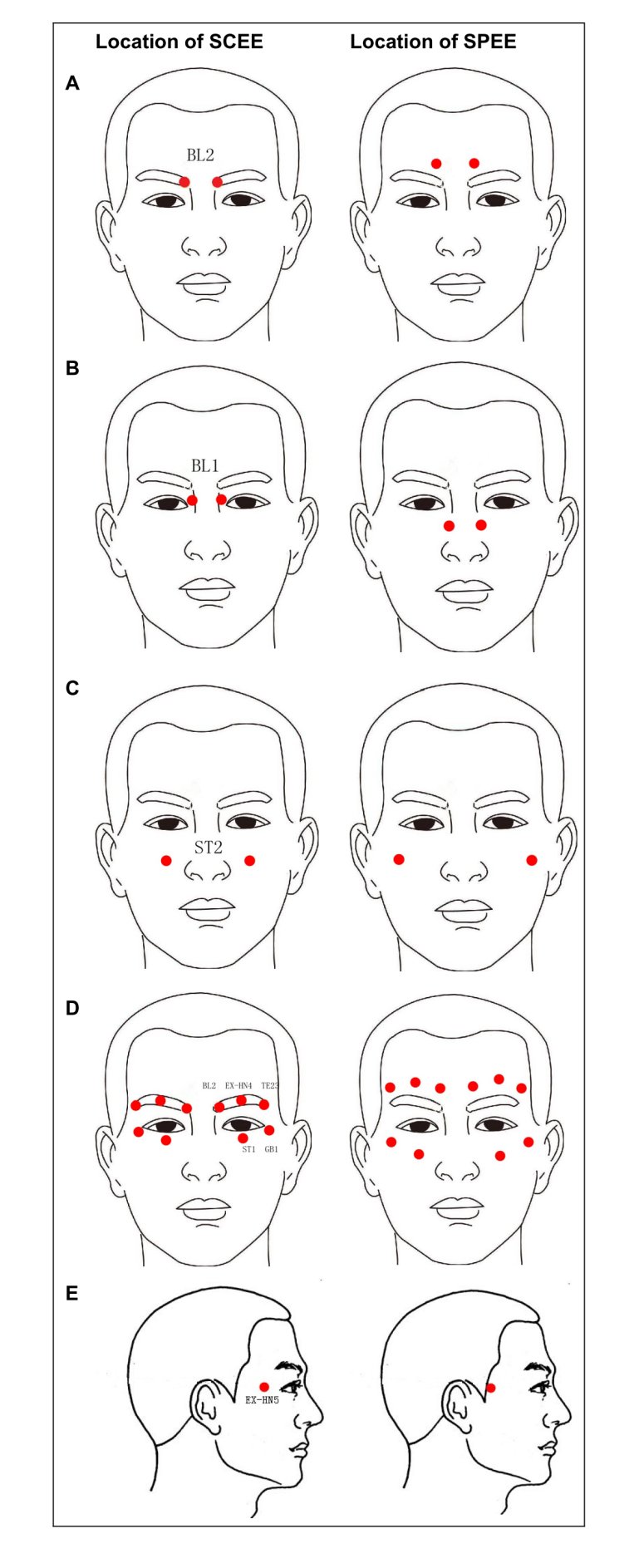


**眼保健操评分标准**

1. 揉天应穴（8分）

|  | 项目 | 评分 | |
| --- | --- | --- | --- |
| 按摩手法 | 是否用大拇指按摩 | 是（1分） | 否（0分） |
|  | 是否用指端按摩 | 是（1分） | 否（0分） |
|  | 按揉手法为画圈 | 是（1分） | 否（0分） |
|  | 按揉直径不超过两个指尖 | 是（1分） | 否（0分） |
| 取穴位置 | 位置在眉头下一点 | 是（1分） | 否（0分） |
|  | 位置在眼眶内侧 | 是（1分） | 否（0分） |
| 按摩力度 | 是否有酸胀感 | 是（1分） | 否（0分） |
| 节奏 | 一拍一圈 | 是（1分） | 否（0分） |

1. 挤按睛明穴（8分）

|  | 项目 | 评分 | |
| --- | --- | --- | --- |
| 按摩手法 | 是否用食指按摩 | 是（1分） | 否（0分） |
|  | 是否用指端按摩 | 是（1分） | 否（0分） |
|  | 按揉手法为先向下按再向上挤 | 是（1分） | 否（0分） |
|  | 按揉直径不超过两个指尖 | 是（1分） | 否（0分） |
| 取穴位置 | 位置位于鼻梁两侧眼角内上半个指尖处 | 是（1分） | 否（0分） |
|  | 按住鼻梁骨，而不是捏住皮肤 | 是（1分） | 否（0分） |
| 按摩力度 | 是否有酸胀感 | 是（1分） | 否（0分） |
| 节奏 | 一拍一圈 | 是（1分） | 否（0分） |

1. 揉四白穴（8分）

|  | 项目 | 评分 | |
| --- | --- | --- | --- |
| 按摩手法 | 是否用食指按摩 | 是（1分） | 否（0分） |
|  | 是否用指端按摩 | 是（1分） | 否（0分） |
|  | 按揉直径不超过两个指尖 | 是（1分） | 否（0分） |
|  | 按揉手法为划圈 | 是（1分） | 否（0分） |
| 取穴位置 | 是否用中指丈量取穴 | 是（1分） | 否（0分） |
|  | 位置是否在眶下孔 | 是（1分） | 否（0分） |
| 按摩力度 | 是否有酸胀感 | 是（1分） | 否（0分） |
| 节奏 | 一拍一圈 | 是（1分） | 否（0分） |

1. 按揉太阳穴轮刮眼眶（16分）

|  |  | 项目 | 评分 | |
| --- | --- | --- | --- | --- |
| 按揉太阳穴 | 按摩手法 | 是否用拇指按摩 | 是（1分） | 否（0分） |
|  |  | 是否用指端按摩 | 是（1分） | 否（0分） |
|  |  | 按揉手法为划圈 | 是（1分） | 否（0分） |
|  |  | 按揉直径不超过两个指尖 | 是（1分） | 否（0分） |
|  | 取穴位置 | 位置为外眼角与眉梢之间向后大约一寸 | 是（1分） | 否（0分） |
|  |  | 位置为凹陷处 | 是（1分） | 否（0分） |
|  | 按摩力度 | 是否有酸胀感 | 是（1分） | 否（0分） |
|  | 节奏 | 1拍1圈 | 是（1分） | 否（0分） |
| 轮刮眼眶 | 按摩手法 | 是否用食指按摩 | 是（1分） | 否（0分） |
|  |  | 是否用第二节指骨关节按摩 | 是（1分） | 否（0分） |
|  |  | 按摩手法为刮 | 是（1分） | 否（0分） |
|  |  | 摩擦眼眶骨性结构 | 是（1分） | 否（0分） |
|  | 取穴位置 | 位置为上下眼眶骨性突出部分 | 是（1分） | 否（0分） |
|  |  | 轮刮长度覆盖整个眼眶 | 是（1分） | 否（0分） |
|  | 按摩力度 | 是否有酸胀感 | 是（1分） | 否（0分） |
|  | 节奏 | 2拍1刮，共4拍 | 是（1分） | 否（0分） |

试验当日，试验组学生的眼保健操将由两位专业中医进行质量控制。

●对照组1：无人指导的眼保健操；

干预前一日，对照组1的两位学生将只接受爱眼知识相关的培训，不参加眼保健操培训，也不与试验组学生有交流和接触。

干预当日，对照组1的两位学生将根据日常习惯做眼保健操。

●对照组2：闭目；

干预前一日，对照组2的两位学生将只接受爱眼知识相关的培训，不参加眼保健操培训，也不与试验组学生有交流和接触。

1. **资料分析和统计方法**

调节反应、近视力、视疲劳得分和瞳孔大小均为连续变量，如果符合正态分布则采用均数和标准差表示，否则采用中位数表达。每一组受试学生做眼保健操前后的各种指标差异采用配对t检验进行分析；三组之间的比较采用单因素方差分析。以P<0.05作为有统计学差异的标准。

问卷 A：请评估你**此刻**的眼部感受，无选择0，非常严重选择10，以此类推。

描述程度（请在0-10上划勾）

|  | 无 | 轻度 |  |  |  | 中度 |  |  |  | 严重 | 非常严重 |
| --- | --- | --- | --- | --- | --- | --- | --- | --- | --- | --- | --- |
| 读书或看近时视物模糊 | 0 | 1 | 2 | 3 | 4 | 5 | 6 | 7 | 8 | 9 | 10 |
| 看远时视物模糊 | 0 | 1 | 2 | 3 | 4 | 5 | 6 | 7 | 8 | 9 | 10 |
| 改变视物距离时（从近到远，或从远到近）再看清困难 | 0 | 1 | 2 | 3 | 4 | 5 | 6 | 7 | 8 | 9 | 10 |
| 眼睛有刺激感或烧灼感 | 0 | 1 | 2 | 3 | 4 | 5 | 6 | 7 | 8 | 9 | 10 |
| 眼睛干涩 | 0 | 1 | 2 | 3 | 4 | 5 | 6 | 7 | 8 | 9 | 10 |
| 眼睛紧张或发紧 | 0 | 1 | 2 | 3 | 4 | 5 | 6 | 7 | 8 | 9 | 10 |
| 头疼 | 0 | 1 | 2 | 3 | 4 | 5 | 6 | 7 | 8 | 9 | 10 |
| 眼睛疲劳 | 0 | 1 | 2 | 3 | 4 | 5 | 6 | 7 | 8 | 9 | 10 |
| 眼睛对明亮光线敏感 | 0 | 1 | 2 | 3 | 4 | 5 | 6 | 7 | 8 | 9 | 10 |
| 眼睛不舒服 | 0 | 1 | 2 | 3 | 4 | 5 | 6 | 7 | 8 | 9 | 10 |
