# Supplementary material for: Efficacy of Chinese Eye Exercises on Reducing Accommodative Lag in School-Aged Children: A Randomized Controlled Trial
Source: PLoS One. 2015 Mar 5;10(3):e0117552. doi: 10.1371/journal.pone.0117552 (PMC4350838; doi:10.1371/journal.pone.0117552)
Supplement: S2 Protocol — (DOCX) [file pone.0117552.s003.docx]

**Protocol for a randomized controlled trial: Efficacy of Chinese eye exercises on reducing accommodative lag in school-aged children**

Shi-Ming Li ,^1^ Meng-Tian Kang,^1^ Xiao-Xia Peng,^2^ Si-Yuan Li,^1^ Yang Wang,^1^ Lei Li,^1^ Jing Yu,^1^ Li-Xin Qiu,^1^ Yun-Yun Sun,^1^ Luo-Ru Liu,^3^ He Li,^3^ Xin Sun^4,5,6^,Michel Millodot,^7^ Ningli Wang, ^1^

Clinicaltrials.gov Registry Number: NCT01756287

^1^ Beijing Tongren Eye Center, Beijing Tongren Hospital, Capital Medical University, Beijing, China

^2^ School of public health, Capital Medical University, Beijing, China

^3^ Anyang Eye Hospital, Henan Province, China

^4^ Chinese Evidence-based Medicine Center, West China Hospital, Sichuan University, China

^5^ Clinical Research and Evaluation Unit, West China Hospital, Sichuan University, China.

^6^ Department of Clinical Epidemiology and Biostatistics, McMaster University, Canada

^7^ School of Optometry and Vision Sciences, Cardiff University, Cardiff, UK

Corresponding author:

Dr. Ningli Wang

Beijing Tongren Eye Center, Beijing Tongren Hospital

No.1, DongJiaoMinXiang, DongCheng District, Beijing

wningli@vip.163.com

**Original protocol**

**Title**: [Efficacy of Chinese Eye Exercises on Reduding Accommodative Lag in School-aged](http://clinicaltrials.gov/ct2/show/NCT01756287?term=chinese+ocular+exercise&rank=1) Children: a Randomized Controlled Trial

**Sponsor**: Beijing Tongren Eye Center, Beijing Tongren Hospital, Capital Medical

University, Beijing, China.

As for a randomized controlled trial on Chinese ocular exercises, only one department is not enough to perform it well. Our group finally included other departments, public school and evidence-based medicine center for study design and statistical analysis (School of public health, Capital Medical University, Beijing, China; Chinese Evidence-based Medicine Center, West China Hospital, Sichuan University, China ), a local hospital to organize the field work (Anyang Eye Hospital, Henan Province, China), and a foreign partner (School of Optometry and Vision Sciences, Cardiff University, Cardiff, UK) to ensure that our study can be well understood by western readers.

**ClinicalTrials.gov Identifier**: NCT01756287

**Study start date**: August 2012

**Estimated study completion date**: November 2013

**Background**:

Myopia is a public health problem worldwide, especially in some Asian countries like China, Singapore, and Japan.[^1^](#_ENREF_1) Chinese eye exercise, originating in 1963 with the theory of Traditional Chinese Medicine (Figure 1-4), is a kind of massage on acupuncture points around the eye to prevent myopia and alleviate visual fatigue.[^2^](#_ENREF_2) The exercise has been spreaded as a community ritual and living habit of primary and high school students for half a century in China. However, the prevalence of myopia in Chinese children increased remarkably in recent years.[^3-5^](#_ENREF_3) Therefore, the efficacy of Chinese eye exercise on preventing myopia or alleviating visual fatigue is widely questioned. Evidence from clinical trials of high level is needed to clarify that whether Chinese eye exercise is effective in slowing and preventing myopia progression, or at least in part in easing the symptoms related to myopia and visual fatigue.[^6^](#_ENREF_6)


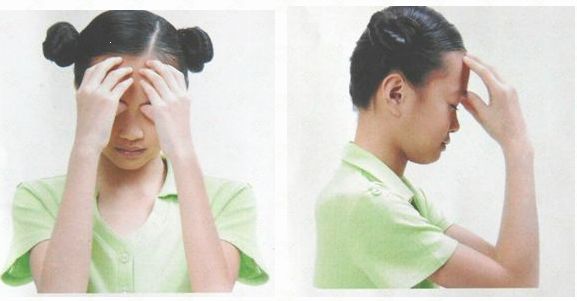


Figure 1. Section 1 of Chinese eye exercises, massage on BL2 (cuanzhu)


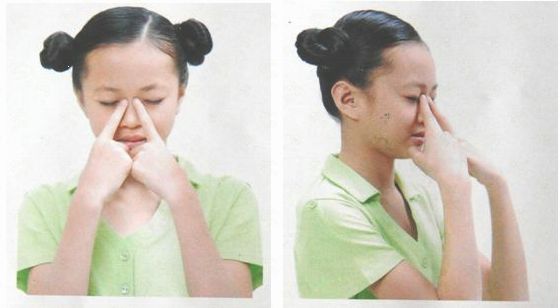


Figure 2. Section 2 of Chinese eye exercises, massage on BL1 (jingming)


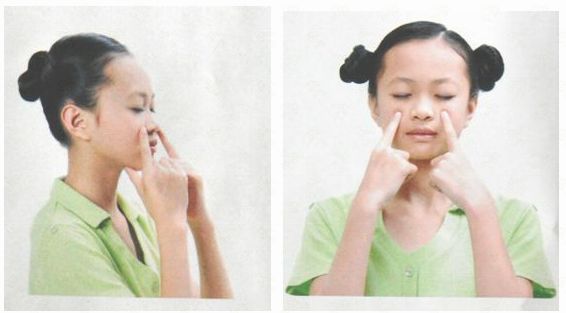


Figure 3. Section 2 of Chinese eye exercises, massage on ST2 (sibai)


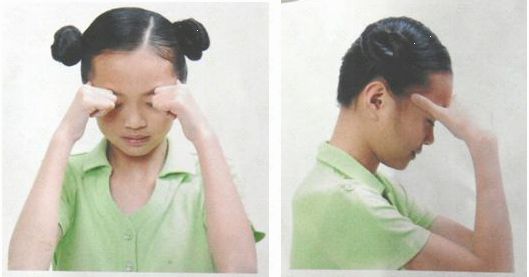


Figure 4. Section 2 of Chinese eye exercises, massage on EX-HN5 (taiyang)

**Purpose**: To assess whether Chinese eye exercise is effective in altering accommodative lag, distant and near visual acuity, and visual symptoms in school-aged children in the short-term.

**Study type**: Interventional

**Study design**: Treatment, Parallel Assignment, Double Blind (Subject, Investigator, Outcomes Assessor), Randomized, Efficacy Study

**Estimated enrollment**: 180

**Eligibility of the participants**

Ages eligible for study: 10 years to 16 years

Genders eligible for study: both

**Inclusion criteria**:

- - 1. Visual acuity: 20/20 or better in each eye;
    2. Spherical error ranging from +0.5 D to -6.0 D and astigmatism less than 1.5 D in each eye, anisometropia less than 1.0 D between the two eyes
    3. No strabismus, amblyopia and any other ocular or systematic diseases that may affect the refractive development

**Inclusion criteria**:

1. Currently using other interventions to control myopia progression (acupuncture, massage, drugs ear needles and so on)
2. Unable to cooperate with the ocular examination and questionnaire survey

**Arms and Assigned Interventions:**

1. **Experimental**: **Standardized Chinese eye exercise**. The participants are trained with standardized Chinese eye exercise which contains accurate position of acupuncture points and appropriate pressure on the points.
2. **Sham comparator: Nonstandardized eye exercise.** The participants are trained with nonstandardized eye exercise performed on wrong positions where no acupuncture points at all.
3. **No intervention:** **Eye closure**. The participants are told to close eyes and don’t do eye exercise at all.

The locations of acupoints of standard Chinese eye exercises (SCEE) and sham point eye exercises (SPEE) are shown in **Appendix 1**.

To guarantee the performance quality of eye exercises, one independent investigator will first demonstrate the procedures of eye exercises and explain the principles for each child. Children will be asked to practice respective exercises under the instruction of the independent investigator for at least three times. During this period, mistakes will be repeatedly corrected until the children perform the eye exercises accurately. Finally, all participants’ performance quality of exercises will be assessed and scored by two independent judges. The assessment covered location, force, scope and frequency of the massage (**See Appendix 2**). Scores greater than 30/40 and differences between two judges less than 5 points were defined as qualified.

**Outcome measures**:

**Primary outcome measures**: accommodative lag before and after the intervention immediately

**Secondary outcome measures**: distant and near visual acuity before and after the intervention immediately

**Other Pre-specified outcome measures**: Self evaluation of visual discomfort score before and after the intervention (**Appendix 3**)

**Statistical analysis**

Data analysis will be performed using SPSS software on an intention-to-treat basis (significance level p < 0.05). Mean differences before and immediately after the interventions will be calculated. Baseline characteristics will be shown as mean ± standard deviation (SD) for continuous data including age, spherical equivalent, corrected distant and near visual acuity, accommodative response, pupil diameter, visual discomfort score and near work load. As for participants’ gender and the proportion of participants with changes, n (%) of male and female and n (%) of participants in each group will be shown as baseline characteristics. Only the results of the right eye were used in the statistical analysis. If the values were normally distributed, analysis of Covariance (ANCOVA) was used otherwise nonparametric test would be used for analysis. Chi-square test was used to compare categorical outcomes.

**References**

1. Pan CW, Ramamurthy D, Saw SM. Worldwide prevalence and risk factors for myopia. Ophthalmic Physiol Opt 2012;32:3-16.

2. Ostberg O, Horie Y, Feng Y. On the merits of ancient Chinese eye acupressure practices. Appl Ergon 1992;23:343-8.

3. He M, Zheng Y, Xiang F. Prevalence of myopia in urban and rural children in mainland China. Optom Vis Sci 2009;86:40-4.

4. He M, Zeng J, Liu Y, Xu J, Pokharel GP, Ellwein LB. Refractive error and visual impairment in urban children in southern china. Invest Ophthalmol Vis Sci 2004;45:793-9.

5. Zhao J, Pan X, Sui R, Munoz SR, Sperduto RD, Ellwein LB. Refractive Error Study in Children: results from Shunyi District, China. Am J Ophthalmol 2000;129:427-35.

6. Wei ML, Liu JP, Li N, Liu M. Acupuncture for slowing the progression of myopia in children and adolescents. Cochrane Database Syst Rev 2011;9:CD007842.

Appendix 1. Locations of acupoints of standard Chinese eye exercises (SCEE) and sham point eye exercises (SPEE)


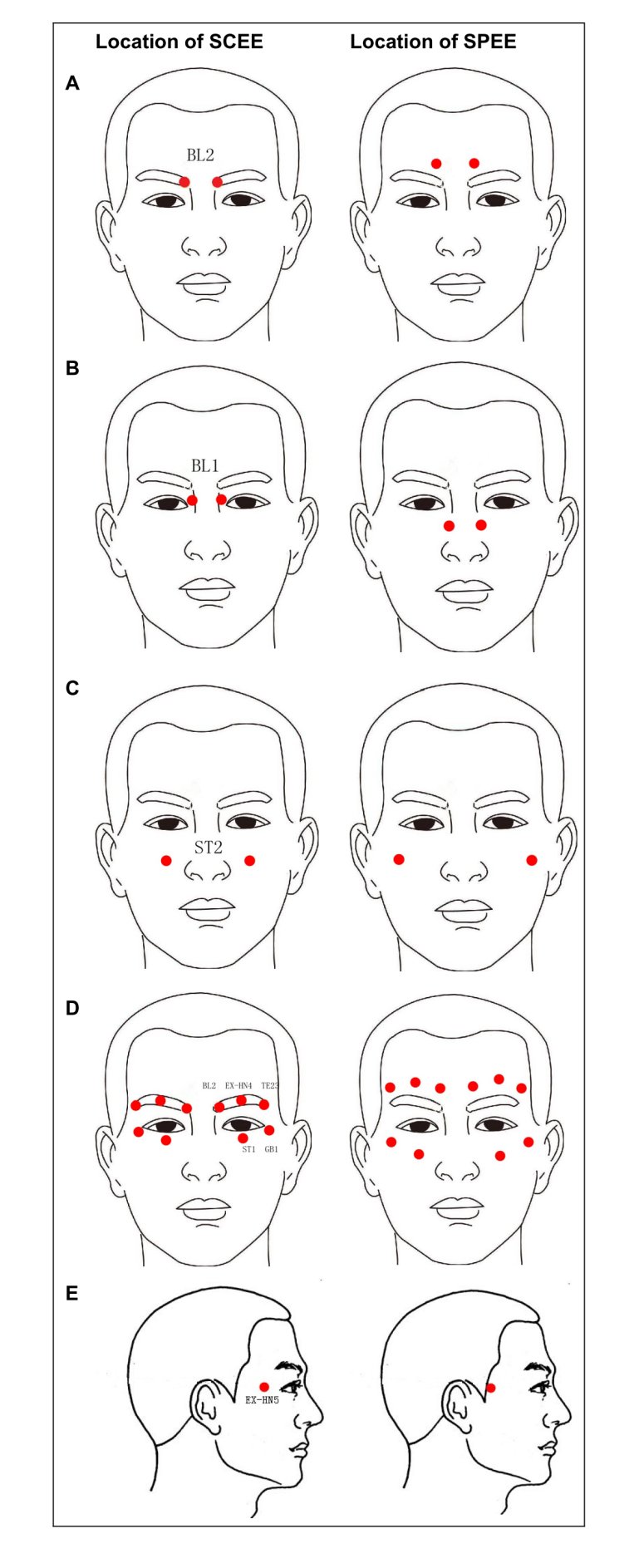


Appendix 2. Point table for evaluating the performance of eye exercises

Part I Press and knead BL2 (cuanzhu) (8 points)

|  | Item | Score | |
| --- | --- | --- | --- |
| Manipulation | Manipulate with thumb | Yes（1 point） | No（0 point） |
|  | Manipulate with fingertip | Yes（1 point） | No（0 point） |
|  | Manipulate in pressing and kneading | Yes（1 point） | No（0 point） |
|  | Manipulation diameter≦2 fingertip | Yes（1 point） | No（0 point） |
| Acupoint Location | At the median end of the eyebrow | Yes（1 point） | No（0 point） |
|  | In the supraorbital notch | Yes（1 point） | No（0 point） |
| Strength Effectiveness | Sense of sore (Qi) | Yes（1 point） | No（0 point） |
| Rhythm | One movement in a beat | Yes（1 point） | No（0 point） |

Part II Press and nip BL1 (jingming) (8 points)

|  | **Item** | **Score** | |
| --- | --- | --- | --- |
| Manipulation | Manipulate with index finger | Yes（1 point） | No（0 point） |
|  | Manipulate with fingertip | Yes（1 point） | No（0 point） |
|  | Manipulate in pressing and nipping | Yes（1 point） | No（0 point） |
|  | Manipulation diameter≦2 fingertip | Yes（1 point） | No（0 point） |
| Acupoint Location | In the depression slightly above the inner canthus | Yes（1 point） | No（0 point） |
|  | Nip bone instead of skin | Yes（1 point） | No（0 point） |
| Strength Effectiveness | Sense of sore (Qi) | Yes（1 point） | No（0 point） |
| Rhythm | One movement in a beat | Yes（1 point） | No（0 point） |

Part III Press and knead ST2 (sibai) (8 points)

|  | **Item** | **Score** | |
| --- | --- | --- | --- |
| Manipulation | Manipulate with index finger | Yes（1 point） | No（0 point） |
|  | Manipulate with fingertip | Yes（1 point） | No（0 point） |
|  | Manipulation diameter≦2 fingertip | Yes（1 point） | No（0 point） |
|  | Manipulate in pressing and kneading | Yes（1 point） | No（0 point） |
| Acupoint Location | Directly below the pupil | Yes（1 point） | No（0 point） |
|  | In the depression of the infraorbital foramen | Yes（1 point） | No（0 point） |
| Strength Effectiveness | Sense of sore (Qi) | Yes（1 point） | No（0 point） |
| Rhythm | One movement in a beat | Yes（1 point） | No（0 point） |

Part IV Press and knead EX-HN5 (taiyang),scrape orbit (16 points)

|  |  | **Item** | **Score** | |
| --- | --- | --- | --- | --- |
| Press and knead EX-HN5 | Manipulation | Manipulate with thumb | Yes（1 point） | No（0 point） |
|  |  | Manipulate with fingertip | Yes（1 point） | No（0 point） |
|  |  | Manipulate in pressing and kneading | Yes（1 point） | No（0 point） |
|  |  | Manipulation diameter≦2 fingertip | Yes（1 point） | No（0 point） |
|  | Acupoint Location | Between the lateral end of the eyebrow and the outer canthus | Yes（1 point） | No（0 point） |
|  |  | In the depression of the infraorbital foramen | Yes（1 point） | No（0 point） |
|  | Strength Effectiveness | Sense of sore (Qi) | Yes（1 point） | No（0 point） |
|  | Rhythm | One movement in a beat | Yes（1 point） | No（0 point） |
| Scrape orbit | Manipulation | Manipulate with index finger | Yes（1 point） | No（0 point） |
|  |  | Manipulate with second phalangeal joint | Yes（1 point） | No（0 point） |
|  |  | Manipulate in scraping | Yes（1 point） | No（0 point） |
|  |  | Scrape bone | Yes（1 point） | No（0 point） |
|  | Acupoint Location | On the bony structures of orbit | Yes（1 point） | No（0 point） |
|  |  | Include the whole orbit | Yes（1 point） | No（0 point） |
|  | Strength Effectiveness | Sense of sore (Qi) | Yes（1 point） | No（0 point） |
|  | Rhythm | One movement in 2 beat | Yes（1 point） | No（0 point） |

Appendix 3. Questionnaire for scoring visual discomfort

Please describe your feeling of eyes . Each question is rated on the following scale and attached a point value as follows:

Describe your feeling

(put √ on the number from 0-10)

|  | **no** | **slight** |  |  |  | **moderate** |  |  |  |  | **severe** |
| --- | --- | --- | --- | --- | --- | --- | --- | --- | --- | --- | --- |
| **Blur at near target** | 0 | 1 | 2 | 3 | 4 | 5 | 6 | 7 | 8 | 9 | 10 |
| **Blur at far target** | 0 | 1 | 2 | 3 | 4 | 5 | 6 | 7 | 8 | 9 | 10 |
| **Have difficulty keeping the target in focus when change the distance (from far to near/near to far)** | 0 | 1 | 2 | 3 | 4 | 5 | 6 | 7 | 8 | 9 | 10 |
| **Burning sensation** | 0 | 1 | 2 | 3 | 4 | 5 | 6 | 7 | 8 | 9 | 10 |
| **Eye dry** | 0 | 1 | 2 | 3 | 4 | 5 | 6 | 7 | 8 | 9 | 10 |
| **Soreness** | 0 | 1 | 2 | 3 | 4 | 5 | 6 | 7 | 8 | 9 | 10 |
| **Headache** | 0 | 1 | 2 | 3 | 4 | 5 | 6 | 7 | 8 | 9 | 10 |
| **Eye stained** | 0 | 1 | 2 | 3 | 4 | 5 | 6 | 7 | 8 | 9 | 10 |
| **Sensitive to light** | 0 | 1 | 2 | 3 | 4 | 5 | 6 | 7 | 8 | 9 | 10 |
| **Eye discomfort** | 0 | 1 | 2 | 3 | 4 | 5 | 6 | 7 | 8 | 9 | 10 |
